# Mechanisms of torsades de pointes: an update

**DOI:** 10.3389/fcvm.2024.1363848

**Published:** 2024-03-05

**Authors:** Yukiomi Tsuji, Masatoshi Yamazaki, Masafumi Shimojo, Satoshi Yanagisawa, Yasuya Inden, Toyoaki Murohara

**Affiliations:** ^1^ Departments of Cardiovascular Research and Innovation, Cardiology and Advanced Cardiovascular Therapeutics, Nagoya University Graduate School of Medicine, Nagoya, Japan; ^2^ Department of Cardiology, Nagano Hospital, Soja and Medical Device Development and Regulation Research Center and Department of Precision Engineering, The University of Tokyo, Tokyo, Japan

**Keywords:** arrhythmia mechanism, animal model of long QT syndrome, torsades de pointes, ventricular fibrillation, electrical storm

## Abstract

Torsades de Pointes (TdP) refers to a polymorphic ventricular tachycardia (VT) with undulating QRS axis that occurs in long QT syndrome (LQTS), although the term has been used to describe polymorphic ventricular tachyarrhythmias in which QT intervals are not prolonged, such as short-coupled variant of TdP currently known as short-coupled ventricular fibrillation (VF) and Brugada syndrome. Extensive works on LQTS-related TdP over more than 50 years since it was first recognized by Dessertennes who coined the French term meaning “twisting of the points”, have led to current understanding of the electrophysiological mechanism that TdP is initiated by triggered activity due to early afterdepolarization (EAD) and maintained by reentry within a substrate of inhomogeneous repolarization. While a recently emerging notion that steep voltage gradients rather than EADs are crucial to generate premature ventricular contractions provides additions to the initiation mode, the research to elucidate the maintenance mechanism hasn't made much progress. The reentrant activity that produces the specific form of VT is not well characterized. We have conducted optical mapping in a rabbit model of electrical storm by electrical remodeling (QT prolongation) due to chronic complete atrioventricular block and demonstrated that a tissue-island with prolonged refractoriness due to enhanced late Na^+^ current (I_Na−L_) contributes to the generation of drifting rotors in a unique manner, which may explain the ECG characteristic of TdP. Moreover, we have proposed that the neural Na^+^ channel Na_V_1.8-mediated I_Na−L_ may be a new player to form the substrate for TdP. Here we discuss TdP mechanisms by comparing the findings in electrical storm rabbits with recently published studies by others in simulation models and human and animal models of LQTS.

## 1 Introduction

Torsades de Pointes (TdP) is a polymorphic ventricular tachycardia (VT) with distinctive fluctuating morphology of QRS complexes that seem to magically twist around the baseline that occurs in congenital and acquired long QT syndrome (LQTS). Although the term has been used to describe polymorphic ventricular tachyarrhythmias in which QT intervals are not prolonged, such as short-coupled variant of TdP currently known as short-coupled ventricular fibrillation (VF) (1, 2) and Brugada syndrome (3), those have different mechanistic and therapeutic entity. TdP is better confined to polymorphic VT with marked QT interval prolongation (4). There have been extensive clinical and experimental studies on LQTS-related TdP [see a scientific statement from AHA/ACC (4) and a comprehensive review paper by Nattel (5)] since it was first described in an elderly patient with heart block by Dessertennes who coined the French term meaning “twisting of the points” (6). Current understanding of the electrophysiological mechanism of TdP is that it is initiated by a triggered beat due to early afterdepolarization (EAD) and maintained by reentry within a substrate of inhomogeneous repolarization. The issue, however, remains attractive because of existence of debates and controversies. Where are premature ventricular contractions (PVCs) that induce TdP originated from? How reentrant sources differ between TdP and other types of polymorphic VT? What predisposes TdP to degeneration into VF?

We have conducted optical mapping in a rabbit model of electrical storm by electrical remodeling (QT prolongation) due to chronic complete atrioventricular block (CAVB) and recently demonstrated that a tissue-island with prolonged refractoriness due to enhanced late Na^+^ current (I_Na−L_) contributes to the generation of drifting rotors in a unique manner, which may explain the undulating QRS morphology characteristic of TdP (7). Moreover, we have proposed that neural Na^+^ channel Na_V_1.8-mediated late Na^+^-current (I_Na−L_) may be a new player to form the substrate for TdP (7). This review article discusses TdP mechanisms by comparing the findings in electrical storm rabbits with recently published studies by others in simulation models and human and animal models of LQTS.

## 2 Consensus of arrhythmia mechanisms in LQTS

EAD-induced triggered activity is associated with both congenital and acquired LQTS, in which arrhythmias occur pause- or bradycardia-dependently. Prolongation of ventricular action potential duration (APD) prolongs the time window during which L-type calcium channel remains open and facilitates the generation of EADs. The channel blocker verapamil eliminates or reduces EADs, abolishes PVCs and suppresses TdP in congenital LQTS type 1 (LQT1) or type 2 (LQT2) caused by mutations in *KCNQ1* or *KCNH2* encoding K_V_7.1 or K_V_11.1 subunit responsible for the slowly or rapidly activating delayed rectifier potassium currents (I_Ks_ or I_Kr_) respectively (8, 9) and in experimental models of LQT1 and 2 (10, 11) as well as type 3 (LQT3) (12) caused by mutations in *SCN5A* encoding sodium channel subunit Na_V_1.5. In addition, there is experimental evidence that some but not all drugs designated as I_Kr_ blockers can generate arrhythmias by augmenting I_Na−L_ (13). Significance of I_Na−L_ in the genesis of arrhythmogenic EADs is supported by the fact that class IB sodium channel blockers lidocaine and mexiletine are effective to suppress TdP in patients with LQT1, 2, and 3 (9, 14).

Delayed afterdepolarization (DAD)-induced triggered activity is also implicated in the mechanisms of LQTS. An increase in cytosolic Ca^2+^ concentrations due to prolongation of action potential causes DADs that are facilitated by rapid pacing and catecholamines. In patients who are mostly children with a much severe form of congenital LQTS, the onset of the arrhythmia is typically not bradycardia- or pause-dependent (15).

Cardiac Purkinje system plays an important role in the onset of TdP. Compared with ventricular myocytes, Purkinje cells are particularly susceptible to EAD and DAD because of the unique cellular electrophysiological property in human Purkinje fibers (16). APD is longer in Purkinje cells than in ventricular myocytes, which is attributed to larger I_Na−L_ and smaller inward rectifier potassium current (I_K1_) (16, 17). Repolarization reserve reduction in LQTS causes more enhanced APD prolongation in Purkinje cells. I_Kr_ blockade with quinidine induces EADs, occurring in 80% of canine Purkinje fibers, but not in ventricular myocardium (18). Focal activity generated in subendocardial Purkinje tissue is the primary trigger of TdP in a canine model of LQT3 (19). Catheter ablation targeting Purkinje potentials is useful in some congenital LQTS patients (20). Lower expression of Na^+^/K^+^-ATPase pump (16), together with larger I_Na−L_, causes intracellular Na^+^ accumulation followed by secondary Ca^2+^-load elevation through Na^+^/Ca^2+^-exchanger, promoting DADs in Purkinje cells. Moreover, Purkinje fibers have 3 times higher transcript expression of *CALM1* encoding calmodulin, a Ca^2+^-binding protein, than ventricular myocardium (16), potentially promoting activation of Ca^2+^/calmodulin-dependent kinase II (CaMKII), a Ca^2+^-activating signal molecule involved in the generation of EAD/DAD (21). Besides Purkinje fibers, EAD-induced triggered activity occurs in myocardium with remarkable APD prolongation. EADs and subsequent TdP events originate from epi- and mid-myocardial layers (10, 22) as well as subendocardial myocardium (12, 23, 24).

Functional reentry underlies the maintenance of TdP. LQTS patients have great dispersion of repolarization regions (25), which favor the occurrence of unidirectional block and reentrant activity. There have been proposals of electrophysiological mechanisms how the undulated QRS axis morphology is formed. El-Sherif and colleagues showed in a canine heart model of LQT3 that a transient bifurcation of the single scroll into 2 simultaneous scrolls that involved both right ventricle and left ventricle (LV) separately (26). Jalife and colleagues, based on the findings in experimental and simulation studies (see the Section 4.5) (27–30), have suggested that a drifting rotor(s) is responsible for TdP maintenance. Since the major laboratories reported in 1990s, however, any information on reentrant activation pattern that produces the specific type of VT has not been added.

## 3 Rabbit model of electrical storm associated with QT prolongation

### 3.1 Rotors anchored by a refractory island drive TdP

**Figure 1:**
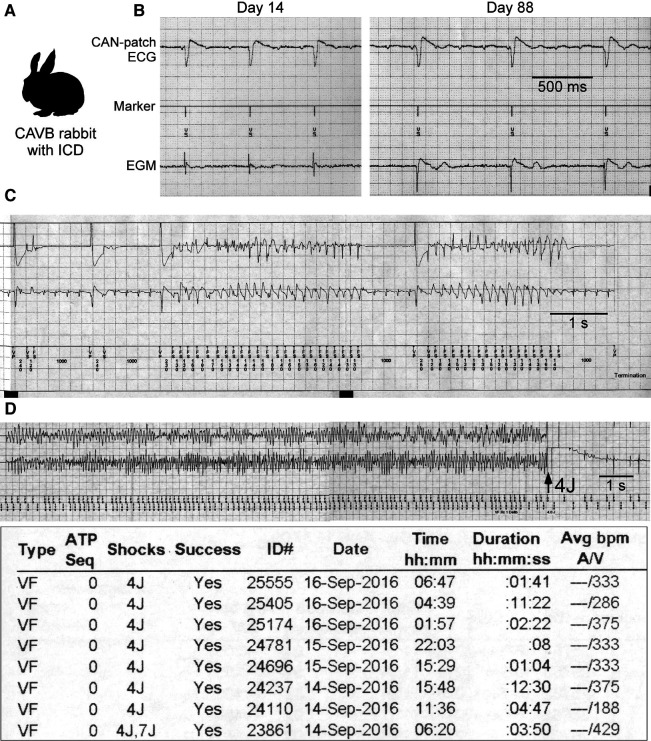
Rabbit model of electrical storm associated with QT prolongation. (**A**) Complete atrioventricular block (CAVB) creation and implantable cardioverter-defibrillator (ICD) implantation are performed. (**B**) CAN-patch electrode ECGs and electrograms (EGMs: 2 leads at the epicardial right ventricle) recorded during ICD interrogation at Day 14 and 88. (**C,D**) Examples of non-sustained VT (NSVT) and ventricular fibrillation (VF) episode and a VF episode report. Modified from Tsuji et al. and Yamazaki et al. (7, 33).

CAVB rabbits show biventricular hypertrophy due to sustained bradycardia and QT prolongation, most of which develop abnormal QTU complex and spontaneous TdP and die suddenly, the electrophysiological features similar to human LQTS (31, 32). When instrumented with implantable cardioverter-defibrillators (ICDs) and followed up for approximately 100 days, the animals have repetitive TdP as non-sustained VT and VF episodes. Electrical storm characterized by clustered VF episodes occurs in ≈50% (Figure 1) (33).

**Figure 2:**
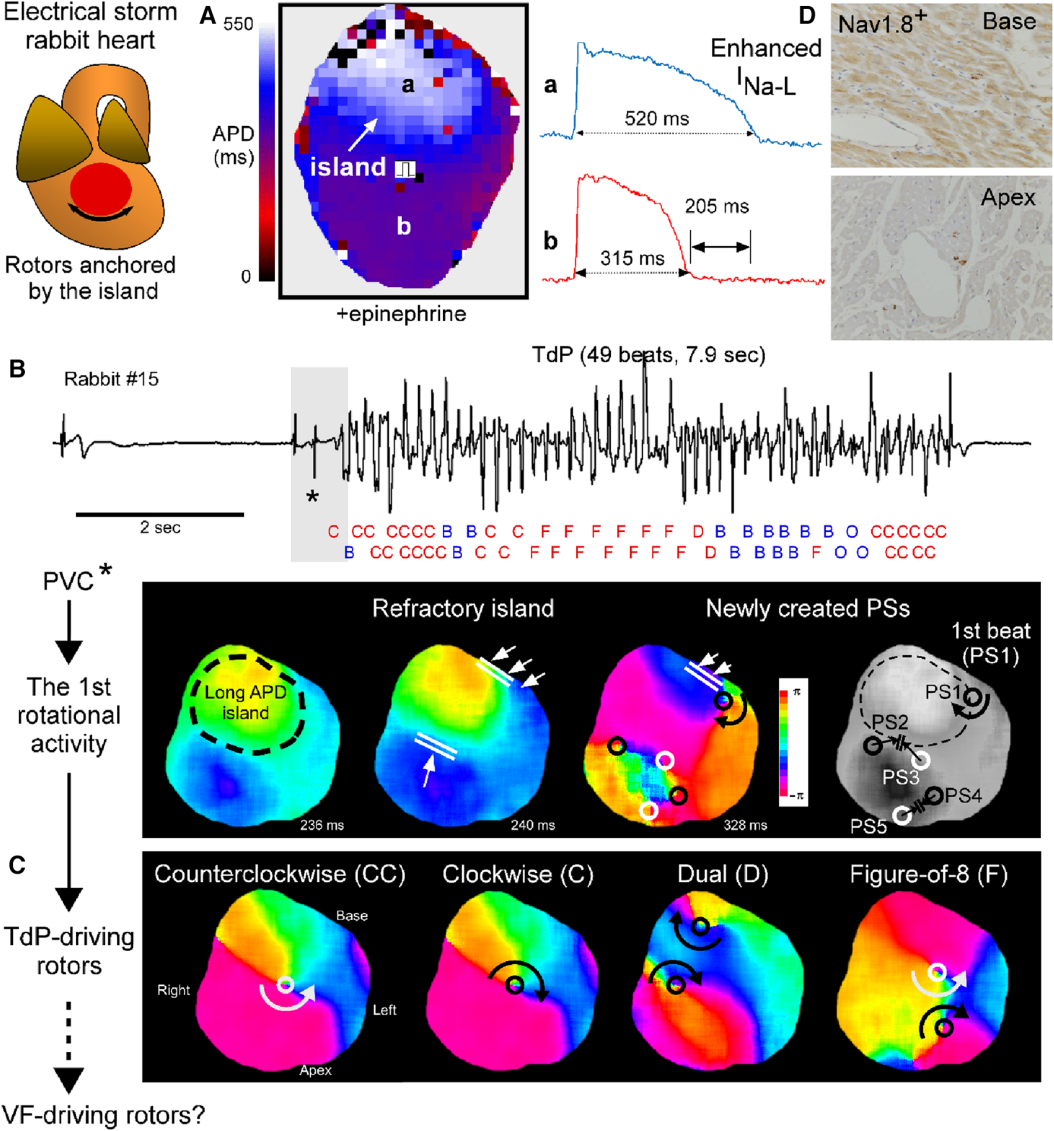
Drifting rotors anchored by a tissue-island drive Tosades de Pointes (TdP). (**A**) Action potential duration (APD) map at a pacing cycle length of 1,000 msec under the perfusate containing 0.1 *μ*M epinephrine in an electrical storm rabbit heart. Values are APD_90_ and APD_90_-dispersion. Regions with prominent APD prolongation look an island. (**B**) A ECG strip of TdP (top) and phase maps for the initiation (bottom) in electrical storm rabbit heart #15. Activation pattern in each of 49 beats analyzed is labelled with C (clockwise rotor), CC (counterclockwise rotor), D (dual rotor), F (figure-of-8), B (breakthrough) or O (one-way propagation). Black/white circle indicate phase singularity (PS, that is, rotor center) with clockwise/counterclockwise rotation. (**C**) Phase maps for rotor-typed activation pattern including CC, C, D and F during TdP. (**D**) Images of immunohistochemistry for Na_V_1.8 at the left ventricular (LV) tissues. Cross-sections of the base and apex in an electrical storm rabbit heart. Na_V_1.8-positive staining in myocardium and nerves at the base, but only nerves at the apex. Modified from Yamazaki et al. (7).

**Figure 3:**
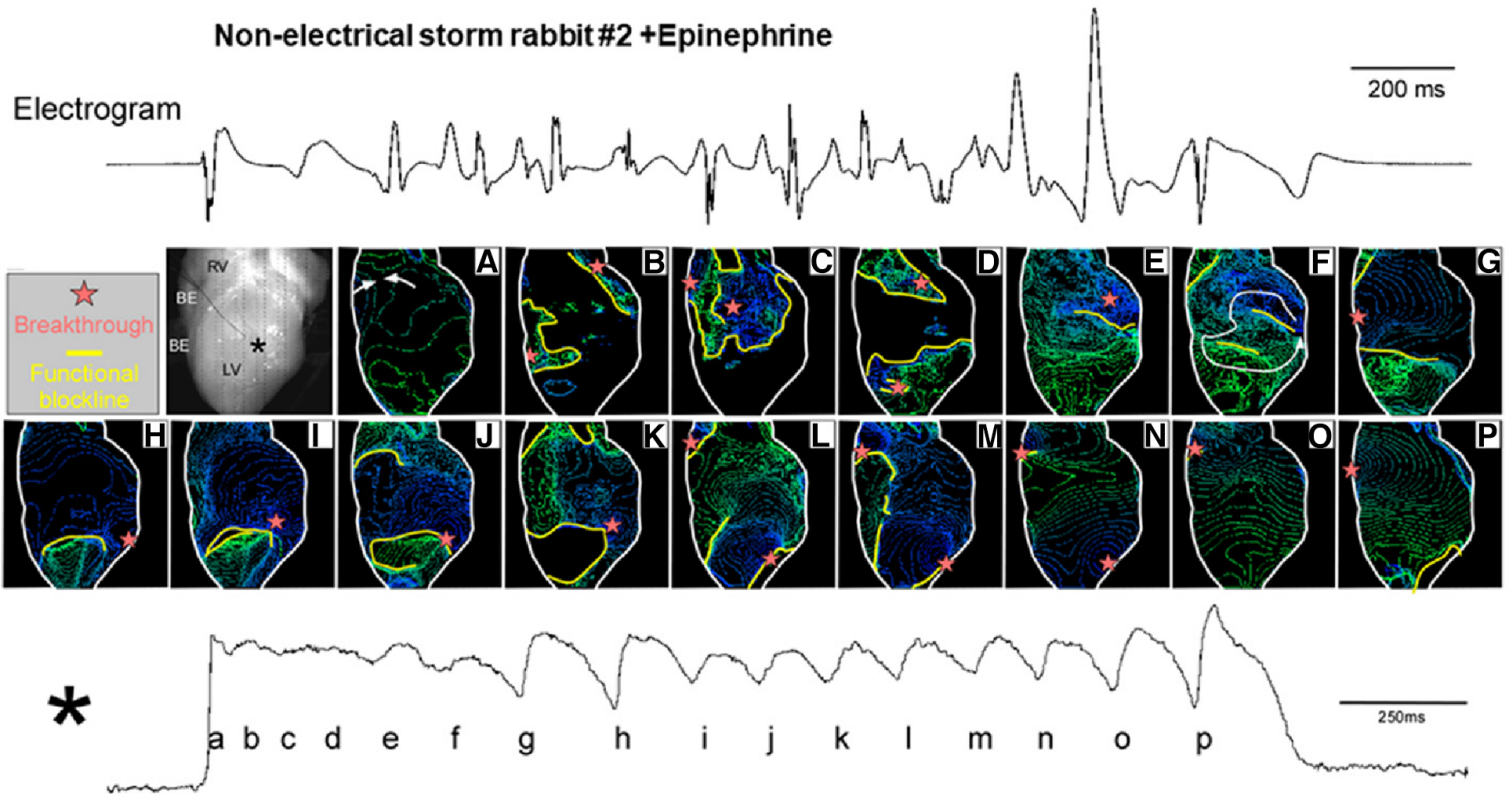
Isochrone maps for non-sustained ventricular tachycardia (NSVT). Mapped heart picture and isochrone maps a-p (upper panel), and the action potential signal at the almost center shown in a black asterisk (lower panel). Isochrone maps for the time points a-p during membrane-voltage oscillation were depicted. Cited from Yamazaki et al. (7).

We have recently demonstrated in a perfused heart from an electrical storm rabbit that drifting rotors anchored by a refractory island drive TdP (7). The findings obtained from this animal model are briefly mentioned here with the illustrated summary (Figure 2).

a. Electrical storm hearts had an island-like regions with long APD at the LV base, leading to increased spatial APD dispersion, which were exacerbated by epinephrine (Figure 2A).
b. A long-lasting TdP event was successfully mapped from the initiation until termination. It was initiated by an exogeneous PVC coupled to an escape rhythm following a long pause that failed to enter the tissue-island with refractoriness and formed 5 reentrant waves at the edge of the island. Four disappeared by mutual annihilation and the remaining 1 rotational activity survived, resulting in the 1st beat of TdP. In addition, the PVC that triggered TdP had a spiky QRS complex, suggesting origination from Purkinje fibers (Figure 2B).
c. Phase map analysis of 49 beats during TdP showed that 32 beats (65%) had rotor typed activation including clockwise, counterclockwise, figure-of-8, and dual rotor, and that the remaining 14 (29%) had breakthrough and 3 (6%) one-way propagation patterns. In 46/49 beats (94%), activation patterns could be determined by mapping at restricted regions: inside the recording area of the anterior epicardial surface. The beat-to-beat intervals of 49 beats were almost identical regardless of the type of activation pattern. Note that rotor centers and breakthrough activations all distributed at the periphery of the island. From these observations, we concluded that 1 or 2 drifting rotor(s) around the island is responsible for TdP perpetuation and that the 17 beats with breakthrough and one-way propagation patterns are likely wavefronts propagating form intramural sources (Figures 2B,C).
d. The localized APD prolongation was attributed to I_Na−L_ enhancement probably due to upregulation of the neuronal Na^+^-channel Na_V_1.8 within myocardium (Figure 2D).
e. The long APD island and great APD dispersion were present in all of 14 electrical storm rabbit hearts optically mapped.
f. APD dispersion *ex vivo* corelated with the number of VF episodes *in vivo*. The association, together with the result of activation patterns of TdP, supports the mechanism described by Jalife and colleagues, whereby either sustained VT or VF results from rapid rotational activity (34, 35). We suggest that the degeneration of TdP to VF occurs when TdP-driving rotors are converted into VF-driving rotors at higher frequency.
g. Another type of a short-lasting non-sustained VT was mapped in a perfused heart from a rabbit without any electrical storm or VF event (Figure 3). Non-electrical storm hearts showed homogeneous APD prolongation with less its dispersion. Multiple breakthrough activations with centrifugal propagation were detected without any reentrant activity. Intriguingly, multiple foci formed membrane voltage oscillation at the center of the recording area (**black asterisk** shown in the heart picture). The findings suggest that multiple foci associated with homogeneous APD prolongation form a relatively benign form of VT, probably corresponding to short-lasting TdP in human LQTS.

### 3.2 Na_v_1.8 may be a candidate for new drug targeting against tachyarrhythmias

**Figure 4:**
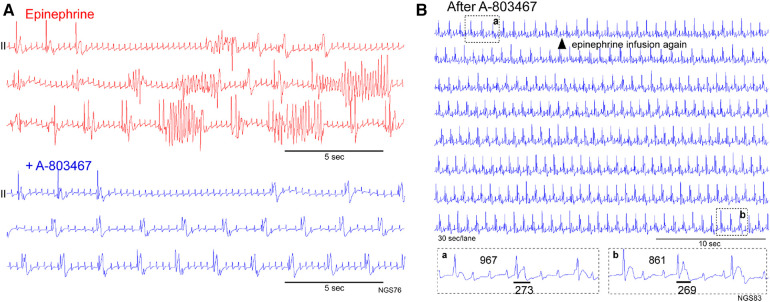
Antiarrhythmic action of the selective Na_V_1.8 blocker A-803467. (**A**) Pause- and bradycardia-dependent TdP appeared during epinephrine infusion (red), but not after intravenous A-803467 injection (blue) in a rabbit at Day 65. (**B**) Epinephrine reinfusion after A-803467 administration in an electrical storm rabbit (Day 56). Epinephrine was infused again from the time point (shown in a filled triangle) in an electrical storm rabbit treated with A-803467. Three-beats ECG traces before (a) and after epinephrine (b) are shown in Boxes. Epinephrine unaffected the QT-interval and did not evoke premature ventricular contraction or ventricular tachyarrhythmia. QT-intervals are shown by horizontal bars.

Na_V_1.8-mediated I_Na−L_ has been implicated as the substrate for TdP in this model. High concentration tetrodotoxin (30 μM)-resistant I_Na−L_ was increased in the LV myocytes and Na_V_1.8 was upregulated in the LV tissues and expressed within myocardium corresponding to the island location in optically mapped electrical storm rabbit hearts, whereas Na_V_1.8-positive nerves were present similarly at the base and apex in electrical storm rabbit tissues (7). Indeed, the selective Na_V_1.8 blocker A-803467 (10 mg/kg, i.v.) attenuated QT prolongation and suppressed TdP evoked by epinephrine in electrical storm rabbits (7). We show here 2 cases supporting the agent to possess antiarrhythmic actions. In a rabbit with 2 VF episodes, epinephrine infusion evoked repetitive TdP pause-dependently when the pacing was turned off, a provocative measure that was ineffective after A-803467 injection (Figure 4A). In another rabbit, epinephrine-reinfusion after treatment with A-803467 did not evoke any ventricular arrhythmia (Figure 4B).

Na_V_1.8 encoded by *SCN10A* is strongly expressed in nociceptive sensory neurons of the dorsal root ganglia and cranial sensory ganglia and much less abundant in hearts than Na_V_1.5 by *SCN5A* that mediates cardiac conduction velocity (36). A series of work following genome-wide association studies provides evidence for Na_V_1.8-mediated I_Na−L_ in mouse and rabbit cardiomyocytes perfused with the selective Na_V_1.8 blocker A-803467 and *SCN10A^−/−^* mice (36, 37). Na_V_1.8 is upregulated in human hypertrophied and failing hearts and A-803467 reduces I_Na−L_, abbreviates APDs and reduces Ca^2+^-spark frequencies and DADs (38, 39). These findings suggest that Na_V_1.8 blockade is a more attractive strategy for treating ventricular tachyarrhythmias than Na_V_1.5-selective I_Na−L_ blockers, preventing arrhythmia without relevant effects on peak I_Na_. Sossalla and colleague has recently reported that Na_V_1.8-driven I_Na−L_ is CaMKII-dependent in human failing cardiomyocytes (40). Since electrical storm is associated with striking CaMKII activation (33), it would be interesting to assess involvement of Na_V_1.8 phosphorylation by CaMKII in enhanced I_Na−L_ in our model.

Some investigators provide mechanistic insights into Na_V_1.8 interaction with Na_V_1.5 in cardiomyocytes. Na_V_1.8 functions as a transcription factor for Na_V_1.5. The C-terminal portion of Na_V_1.8 encoded by *SCN10A-short* transcripts which appears to be genetic variants in and around *SCN10A*, increases Na_V_1.5 expression and channel current in mouse cardiomyocytes (41). In culture cells, the Na^+^ current increases 2-fold in HEK cells coexpressing wild type *SCN10A* and *SCN5A*, compared to those expressing *SCN5A* alone, and the Na^+^ current decreases in HEK cells coexpressing *SCN10A* with Brugada mutation and wild type *SCN5A* (42). Further studies are needed in our model to investigate whether Na_V_1.8 overexpressed in electrical storm hearts is a full-length or a C-terminal portion.

While the potential targeting of Na_V_1.8 against ventricular arrhythmias in failing and hypertrophic hearts is intriguing, the expression and function of Na_V_1.8 in structurally normal hearts, where TdP can occur, remain debatable. More studies would be highly required to clarify whether and how Na_V_1.8 blockade with A-804367 affects peak and late components of Na^+^ currents in myocytes from electrical storm rabbits and other models of LQTS.

## 4 Relationship to previous studies in related animal models and patients

**Figure 5:**
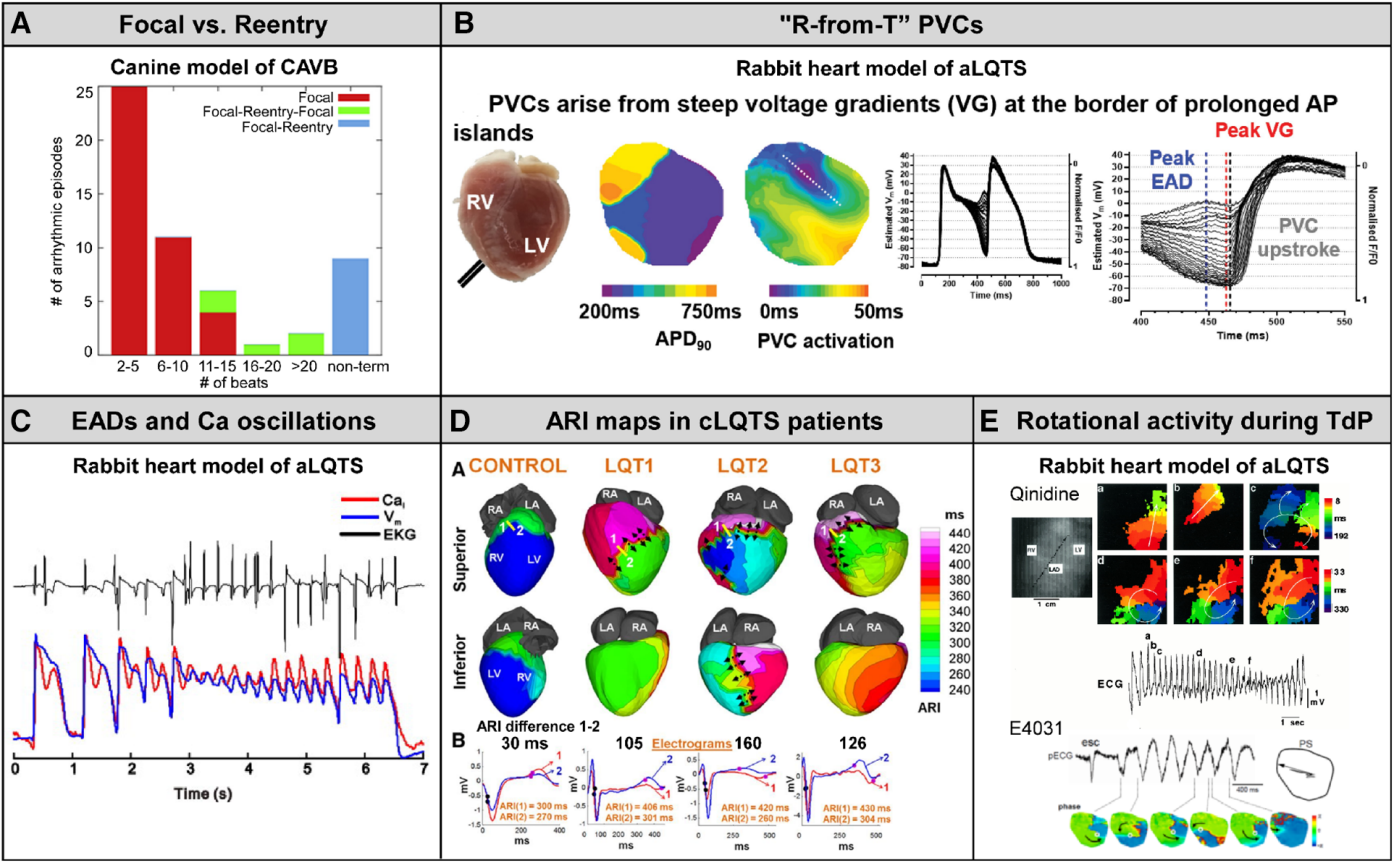
The key findings in related animal models and congenital LQTS patients. (**A**) Canine model of chronic complete AV block (CAVB). Nonterminal episodes of TdP are always maintained by reentry. Cited from Vandersickel et al. (43). (**B**) An emerging concept “R-from-T” verified in normal rabbit hearts under the condition of QT interval prolongation (E-4031 0.5 *μ*M and 50% K^+^/Mg^2+^). Cited from Alexander et al. (44). (**C**) Burst of EADs coupled to Ca^2+^ oscillation form TdP in normal rabbit hearts under the condition of QT interval prolongation (bradycardia, dofetilide 0.5 *μ*M and reduced K^+^/Mg^2+^). Cited from Nemec et al. (11). (**D**) Maps of activation recovery interval (ARI), surrogate maker for action potential duration, in genotyped congenital LQTS syndrome (LQT1, LQT2 and LQT3). Cited from Vijayakumar et al. (25). (**E**) Rotational activity during TdP detected in normal rabbit hearts under the condition of QT interval prolongation (top: quinidine 5 *μ*M and reduced K^+^, bottom: E-4031 0.5 *μ*M and reduced K^+^/Mg^2+^). Cited from Asano et al. and Maruyama et al. (27, 45).

Studies in related animal models and patients with congenital LQTS are briefly reviewed here. The key findings are shown in Figure 5.

### 4.1 Canine model of CAVB

Spatial dispersion of repolarization has been implicated as a substrate of TdP in CAVB dogs. In studies by Vos and colleagues (46, 47), using 56 needle electrodes distributed transmurally to record 224 unipolar electrograms simultaneously, TdP was initiated at the site with maximal heterogeneity of repolarization in the LV and the selective I_Na−L_ inhibitor GS967 abolishes TdP through reduced spatial dispersion of repolarization. These findings in CAVB dogs are supported by our results showing that the first beat of TdP following a triggering PVC was a reentrant wave arising in the periphery of the long APD island (Figure 2). In addition, Vos's group originally reported in CAVB dogs that more than 90% of the beats during TdP were focal (48), but a most recent study by his collaborators has developed a novel methodology to detect reentry loops by using graph search algorithms and elegantly demonstrated that TdP can be driven by focal activity as well as reentry depending on the duration of the episode: longer-lasting TdP is maintained by reentry (Figure 5A) (43). Nonterminal TdP that lasts >10 s was all perpetuated by reentry.

### 4.2 A unifying mechanism of trigger and substrate in simulation models

A mixture of focal activity and reentry has been reported to underlie EAD-mediated arrhythmias in rat/rabbit hearts exposed to H_2_O_2_ (49) or hypokalemia in the absence or presence of dofetilide (50) and a transgenic LQT2 rabbit model (51) as well as in the rabbit ventricle simulation model (52). Repetitive focal activations from single or multiple foci from EAD zones, together with regional APD gradients by areas with EADs next to regions without EADs leading to localized conduction block and initiation of reentry, account for the combination of mechanisms underlying EAD-induced TdP and polymorphic VT (53). Moreover, recent simulation studies (54), together with experiments in transgenic LQT2 rabbit hearts (51) and normal rabbit hearts during conditions with I_Kr_ blockade with E4031 (0.5 *μ*M) plus 50% K^+^/Mg^2+^ (44), have strengthened the idea that steep APD gradients rather than EADs give rise to both the triggering PVC and the substrate for reentrant tachyarrhythmia, the mechanism termed “R-from-T” (Figure 5B) (54). The TdP event we detected in an electrical storm rabbit heart was triggered by a PVC via classical “R-on-T” mechanism in which an exogenous PVC encounters a refractory region (Figure 2), but not “R-from-T”. Since the number of tachyarrhythmia events optically mapped and analyzable was limited, we could not detect ventricular tachyarrhythmias with the “R-from-T” initiation mode. Some PVCs certainly originated at regions adjacent to the long APD island in electrical storm hearts, suggesting that “R-from-T” may be involved in TdP in this model. However, roles of “R-from-T” vs. “R-on-T” in arrhythmogenicity remain unclear.

### 4.3 Rabbit heart models of acquired LQTS

There are differences in the observed phenomena at the onset and maintenance of arrhythmias between rabbit models. Studies in normal rabbit hearts subjected to the condition of QT interval prolongation by bradycardia, I_Kr_ blockade with E-4031 or dofetilide and reduced K^+^/Mg^2+^ showed that bursts of EADs coupled to Ca^2+^ oscillations near the base (area of long APD) occurring in zones as small as 1 mm^2^ correspond to TdP (Figure 5C) (11, 55). In electrical storm rabbit hearts, EADs were observed in 5/14 electrical storm hearts and some PVCs occurred from phase 2 of the preceding action potential in electrical storm hearts, but neither reentrant excitation wavefronts propagating from the phase 2 PVC nor a burst of EADs were detected. Membrane voltage oscillation resulting from multiple foci in a non-electrical storm rabbit heart with homogeneous APD prolongation (Figure 3) is an etiology different from bursts of EADs observed in those models (11, 55). The high concentration (0.5 *μ*M) of E-4031 or dofetilide used in the acute model produces prominent APD prolongation, to an extent much greater than that occurring spontaneously in our chronic model, possibly explaining some of the discrepancy. A recently published study in a guinea-pig surrogate model of LQT3 demonstrating that voltage/calcium uncoupling predates sustained ventricular tachyarrhythmia provides evidence of reentrant excitation responsible for TdP potentially degenerating into VF (56).

### 4.4 ECGi mapping in patients with congenital LQTS

Spatially heterogeneous prolongation of activation recovery interval (ARI), a surrogate marker of APD, of ventricular epicardium has been demonstrated in genotyped LQTS patients, using non-invasive ECG imaging (ECGi) technique (25). Repolarization gradients resulting from the localized ARI prolongation are found steeper in patients with than without history of syncope or sudden cardiac arrest and ICD-defibrillation (Figure 5D). However, ionic mechanisms of heterogeneous ARI prolongation in congenital LQTS, the syndrome monogenic but with variable phenotypes, remain unclear. The findings in electrical storm rabbits agree with the human data: the boundary of the long APD island likely corresponds to regions with steep repolarization gradients. Our proposal of Na_V_1.8-mediated I_Na−L_ enhancement provides insights into the pathophysiology of LQTS. Acquired regulation/modification of cardiac Na^+^-channels by Na_V_1.8 may be one of the important factors involved in the heterogeneous substrate in LQTS.

### 4.5 Drifting rotors with a unique behavior may produce undulating QRS morphology

Studies in simulation models have reported that the polymorphism of VT in the ECG, including TdP, can be determined by a Doppler effect resulting from drift of a single spiral wave (28–30). In agreement with the modeling studies, some investigators reported a meandering spiral wave in normal rabbit hearts subjected to hypokalemia plus quinidine (27) or dofetilide (45) and in a transgenic LQTS type-2 rabbit model (57), but rotational activation was detected in only a few beats during TdP (Figure 5E) and none one has characterized the dynamics of spiral waves. Our study in electrical storm rabbits is the first to report drifting rotors in a unique manner. The detection of a clockwise, counterclockwise, figure-of-8 and dual rotor, all whose cores swirl in the periphery of the long APD island, suggests 1 or 2 scroll source(s) with various types of filaments, potentially contributing to undulating QRS axis formation.

## 5 Future direction

Overall, the findings from clinical and experimental studies currently available suggest that a PVC arising from steep repolarization gradients (R-from-T) or remote regions (R-on-T) propagates unidirectionally toward regions with abnormally delay repolarization termed a refractory island, resulting in the generation of rotational activity responsible for the 1st beat of TdP followed by drifting rotors around the island that perpetuate TdP. The long-lasting TdP-driven rotors has potential to degenerate into VF. Future studies are needed to characterize the 3-D structure as well as to explore the electrophysiological and molecular features of the torsadogenic island.

Our attention moves to the issue on why electrical storm rabbits have liability to VF. According to the mother rotor hypothesis, APD shortening and increased excitability stabilize rotors at higher frequency (58). Ion channels potentially involved in the conversion from TdP- to VF-driving rotors might include the ATP-sensitive K^+^ (I_K−ATP_) channel, Ca^2+^-activated potassium (SK) channel and I_K1_ channel. TdP can manifest as acutely decreased pump function and hemodynamic instability (5), leading to myocardial ischemia and ATP depletion that activates I_K−ATP_ channel. In ICD patients with ischemic or non-ischemic cardiomyopathy, myocardial ATP depletion detected by phosphorous magnetic resonance spectroscopy is a predictor for appropriate ICD therapies against VT/VF (59). Preliminary data on proteome in electrical storm heart tissues shows that VF storm is associated with mitochondrial dysfunction and oxidative phosphorylation inactivation (unpublished). In failing rabbit hearts, SK channel activation underlies the post-shock APD shortening leading to recurrent VF (60). In cardiomyocytes from CaMKII-overexpressing rabbits and CAVB rabbits show an increase in I_K1_ (31, 61). Future studies are needed to identify and characterize VF-driving rotors as well as to explore molecular mechanisms by which the conversion is promoted in a rabbit model of electrical storm, which might open a new avenue to develop novel therapeutic strategies to VF.
